# Gastric mucosa-associated lymphoid tissue (MALT) lymphoma: clinicopathological study and treatment outcome in 50 patients

**DOI:** 10.11604/pamj.2020.37.372.27094

**Published:** 2020-12-23

**Authors:** Yosr Zenzri, Lamia Charfi, Ghada Sahraoui, Yosra Yahyaoui, Karima Mrad, Nadia Boujelbene, Raoudha Doghri

**Affiliations:** 1 Pathology Department, Salah Azaiez Institute, Tunis, Tunisia,; 2 Medical Oncology Department, Salah Azaiez Institute, Tunis, Tunisia

**Keywords:** Mucosa-associated lymphoid tissue, lymphoma, prognosis, treatment

## Abstract

The stomach is the most frequent site of extranodal lymphoma. Mucosa-associated lymphoid tissue (MALT) lymphoma is a low-grade, B-cell neoplasm strongly associated with Helicobacter pylori (HP) infection. The presenting complaints of gastric MALT lymphoma are usually nonspecific. HP eradication is regarded as the first-line therapy in early stage disease. Management of patients who failed to achieve remission following HP eradication include chemotherapy, radiotherapy and in selected cases, surgery. The aim of the present study was to examine the clinical characteristics and treatment outcome of patients with gastric MALT lymphoma.

## Introduction

Mucosa-associated lymphoid tissue (MALT) lymphomas are extra-nodal B-cell marginal zone lymphomas that mainly follow an indolent course. It accounts for nearly 50% of primary gastric lymphomas. It is often associated with *Helicobacter pylori* (HP) infection. Persistent infection with HP causes chronic gastritis and is considered as a major risk for gastric MALT lymphoma [1]. The diagnosis of gastric MALT lymphoma is based on endoscopic and histopathological features. This study was undertaken to evaluate the clinicopathological characteristics and treatment outcome of patients with gastric MALT lymphoma.

## Methods

Our study was respectively carried out over a 17 year period (2001-2018) and conducted at our pathology department (Salah Azaïz Institute, Tunisia). Fifty cases were systematically reviewed. Clinical information for each patient including age, gender, medical history, clinical presentation and Ann Arbor stage was abstracted from the medical records. Additional data recorded including the performance status and the endoscopic features were obtained. Histological evaluation of post-treatment biopsies was performed according to the “Groupe d´Etude des Lymphomes de l´Adulte” (GELA) grading system [2]. The data were analyzed on the statistical package for the social sciences (SPSS) version 20. Univariate comparisons between groups were performed using chi-squared tests for dichotomous variables or Fisher´s test when appropriate. P<0.05 was considered to be significant. Cumulative event rates were calculated using the method of Kaplan-Meier.

## Results

The mean age was 51 years. Characteristics of gastric MALT lymphoma are summarized in Table 1. The most common presenting symptoms included epigastric pain (82%) and vomiting (34%). The disease was most often localized in the antrum (n=39). The histologic appearance was characterized by: lymphoepithelial lesions (82%), scattered large cells (n=18) and lymphoplasmacytic differentiation (n=10). HP was detected in 44 patients. The presence of HP infection was confirmed in all cases by giemsa stain in the gastric biopsies.

**Table 1:** characteristics of gastric MALT lymphoma: univariate analysis of the overall survival

Characteristics	Number	Percentage	P value
**Sex**			
Male	27	54%	0.5
Female	23	46%	
**Family history of gastric cancer**			
Yes	4	8%	-
No	46	92%	
**Performance status**			
0-1	40	80%	0.004
2-3	10	20%	
**LDH**			
Elevated	13	26%	0.7
Normal	37	74%	
**Ann arbor stage**			
IE-IIE	33	66%	0.036
IIIE-IVE	17	34%	
**Response to initial treatment**			
Yes	19	37%	0.05
No	27	63%	
**HP**			
Yes	44	88%	0.3
No	6	12%	
**GELA (at the end of treatment)**			
CR/MRD	30	65%	0.005
rRD/NC	16	35%	

LDH=lactate dehydrogenase; HP=*helicobacter pylori*; CR/MRD=complete remission/minimal residual disease; rRD/NC=residual disease/no change

Four patients underwent surgery, 17 had chemotherapy (4 had rituximab-chlorambucil and 13 had R-CHOP21), 25 had received HP eradication therapy. Four patients were lost to follow-up and didn´t receive any treatment. HP eradication was obtained in 18 patients. Sixty nine percent of patients achieved complete remission or minimal residual disease. Residual disease and no change were noted in 3% and 28% of patients, respectively. Complete remission or minimal residual disease in HP (+) gastric MALT lymphoma were noted in 60% patients. There was no association between HP status and GELA response (p=0.7). Histologic transformation to large-cell lymphoma was noted in 8 patients. An association between HP status and transformation to large-cell lymphoma was observed (p=0.002). One patient developed a gastric adenocarcinoma. The 5-years overall survival was 83%. At the end of follow-up, 30 patients achieved complete remission, 8 patients died, 8 patients had progressive disease and 4 patients were lost to follow-up. Good performance status, stage IE-IIE, response to initial treatment and complete remission/minimal residual disease according to GELA grading system were significantly associated with prolonged survival after treatment (p=0.004; 0.036; 0.05 and 0.005, respectively).

## Discussion

According to some epidemiologic studies, gastric MALT lymphomas are slightly more common in males than in females as our results show [3]. This neoplasm occurs over a wide age range with a mean of 57 years [4]. The clinical presentation is similar to gastric carcinomas and benign ulcers and commonly includes abdominal pain or discomfort. The disease was most often localized in the antrum. This is in accordance with previous reports [5]. Most patients are diagnosed at an early stage as our results show. Identification of the etiologic role of HP infection has radically changed the therapeutic approach for this disease. Eradication therapy is the standard treatment for gastric MALT lymphoma. Surgery is reserved for complications such as perforation or uncontrollable bleeding [5]. For patients who failed to achieve remission following HP eradication, chemotherapy could be used. Rituximab and oral alkylating agents (chlorambucil, cyclophosphamide) or purine nucleoside analogs (cladribine, fludarabine). Chemotherapy regimen which contains anthracycline should be used in patients with massive tumoral masses [6]. In our study, 17 patients had chemotherapy. Different studies suggest that HP infection plays a relevant role even in the high-grade, diffuse large B-cell lymphomas of the stomach [4]. An association between HP status and transformation to large-cell lymphoma was observed in the present study (p=0.002). In our study, the 5-year overall survival was 83%. This rate was lower than in the previously published clinical series where this rate exceeded 85% [7]. Age >60 years, advanced stage, poor performance status and elevated LDH were associated with poor outcome in others series [8].

## Conclusion

Based on our results, variables associated with decreased survival were: advanced stages and poor performance status. Response to initial treatment and complete remission/minimal residual disease according to GELA grading system were significantly associated with prolonged survival.

### What is known about this topic

- Mucosa-associated lymphoid tissue (MALT) lymphoma is often associated with Helicobacter pylori infection;
- Eradication therapy is the standard treatment for gastric MALT lymphoma;
- Chemotherapy could be used for patients who failed to achieve remission following Helicobacter pylori eradication.

### What this study adds

- There was no association between Helicobacter pylori status and GELA response;
- An association between Helicobacter pylori status and transformation to large-cell lymphoma was observed in our study;
- Good performance status, stage IE-IIE, response to initial treatment and complete remission/minimal residual disease according to GELA grading system were significantly associated with prolonged survival after treatment.
